# ADVERSE EVENTS IN OLDER ADULTS AT RISK FOR DEMENTIA DURING VIRTUAL EXERCISE AND COGNITIVE TRAINING

**DOI:** 10.1093/geroni/igad104.2937

**Published:** 2023-12-21

**Authors:** Karla Faig, Molly Gallibois, Alanna Bohnsack, Chris McGibbon, Andrew Sexton, Josée Haché, Samantha Knill, Pamela Jarrett

**Affiliations:** Horizon Health Network, Saint John, New Brunswick, Canada; University of New Brunswick, Fredericton, New Brunswick, Canada; Horizon Health Network, Saint John, New Brunswick, Canada; University of New Brunswick, Fredericton, New Brunswick, Canada; University of New Brunswick, Fredericton, New Brunswick, Canada; Vitalité Health Network, Moncton, New Brunswick, Canada; Horizon Health Network, Saint John, New Brunswick, Canada; Horizon Health Network, Saint John, New Brunswick, Canada

## Abstract

Synchronizing Exercises, Remedies in Gait and Cognition at Home (SYNERGIC@Home/SYNERGIE~Chez soi) is a home-based, double-blind, randomized controlled trial (NCT04997681) targeting community-dwelling older adults at risk for dementia. Anglophone and Francophone participants living in an Atlantic Canadian province (New Brunswick) underwent 16 weeks (three sessions/week) of interventions which consisted of virtual cognitive and physical exercise designed to delay or prevent the onset of dementia. This research aims to determine the frequency, severity, and relationship of the adverse events (AEs) that occurred during the intervention phase. All reported AEs were reviewed and managed by members of the research team, including a geriatrician. Participant’s (n=60) mean age was 68.9 years (SD 6.58), and the majority (76.7%) were female. Forty percent were living in rural communities, and 60% were urban. Of these participants, there was a total of 88 AEs reported by 42 individuals; 70% having at least one AE. The most common AE (39.8%) was musculoskeletal in nature. The majority (71.6%) of all AEs were unrelated to the intervention, and 69.3% of all AEs were classified as mild. There was only one serious AE, and it was unrelated to the intervention. The intervention had to be discontinued in two individuals, and modifications were required in 59 cases for participants to continue in the study. When delivered virtually with videoconference supervision, physical and cognitive interventions resulted in no serious related AEs and few related, mostly mild AEs, that were safely managed through modifications to the physical interventions.

